# Gut-bone axis response to dietary replacement of soybean meal with raw low-tannin faba bean seeds in broiler chickens

**DOI:** 10.1371/journal.pone.0194969

**Published:** 2018-03-28

**Authors:** Ewa Tomaszewska, Piotr Dobrowolski, Renata Klebaniuk, Małgorzata Kwiecień, Agnieszka Tomczyk-Warunek, Sylwia Szymańczyk, Sylwester Kowalik, Anna Milczarek, Tomasz Blicharski, Siemowit Muszyński

**Affiliations:** 1 Department of Animal Physiology, University of Life Sciences in Lublin, Lublin, Poland; 2 Department of Comparative Anatomy and Anthropology, Maria Curie-Skłodowska University, Lublin, Poland; 3 Department of Bromatology and Food Physiology, University of Life Sciences in Lublin, Lublin, Poland; 4 Department of Animal Nutrition and Feed Management, Siedlce University of Natural Sciences and Humanities, Siedlce, Poland; 5 Chair and Department of Rehabilitation and Orthopaedics, Medical University in Lublin, Lublin, Poland; 6 Department of Physics, University of Life Sciences, Lublin, Poland; University of Connecticut, UNITED STATES

## Abstract

It seems that faba bean (FB) seeds could be a good protein-energy component in animal feed, but the presence of anti-nutritional substances limits their use as a substitute of soybean meal. The aim of the study was to examine the influence of different concentrations of raw, low-tannin, FB seeds on the gut-bone axis in Ross 308 broilers. One-day old chickens were randomly subjected to one of the 3 dietary treatments: the control group was fed standard diet based on soybean meal and without FB seeds, and two groups were fed 8%/15% and 16%/22% of raw low-tannin FB seeds in the starter and grower, respectively. On the 35^th^ day, hematological and serum biochemical analyses as well histomorphometry of the small intestine and liver tissue and bone mechanical tests were performed. The diet type had no effect on the body weight gain and feed conversion ratio. However, the basal intestinal structures were significantly reduced in birds fed the lower concentration of FB. The enlargement of nerve plexuses was dependent on the concentration used in the diet and, additionally, on the kind of plexus and location in the intestinal tract. The liver was characterized by an increase in non-hepatocytes. There was no influence of the low-tannin FB seeds on most of the analyzed serum parameters in the 35-day-old broiler chickens, except the decreased concentration of total cholesterol and Ca in both experimental groups, triglycerides in group I, and P and uric acid in group II. Furthermore, the increasing concentration of the dietary low-tannin FB did not influence the activities of AspAT (except the group fed the higher amount of FB), ALAT, and LDH. The broiler chickens had no visible leg lesions and no problem in the locomotor function, but the tibiae were lighter mainly in birds fed the higher concentration of FB seeds. Geometric analysis revealed reduction of the cross section area and wall thickness, indicating a decline in the bone midshaft, which influenced the densitometric parameters and the results of mechanical tests. In conclusion, the dietary inclusion of raw low-tannin FB seeds had no negative effects on broiler growth, but disturbed the intestine structure and tibia characteristics. Therefore, all these negative effects necessitate additional examinations before inclusion of raw low-tannin FB seed into poultry diet.

## Introduction

The use of genetically modified soybean prompts a search for protein sources that could be alternatively introduced in animal diet [1]. Faba bean seeds (FB; *Vicia faba* L. *minor*) can be a good protein-energy component in monogastric animal feeding, including poultry, due to their chemical composition: high protein content (26–30%) and a high concentration of starch (40%) [2]. On the other hand, a negative feature of FB seeds is the presence of anti-nutritional substances such lectins, alkaloids, saponins, phytic acid, non-starch polysaccharides, enzyme inhibitors, and condensed polyphenols (tannins), which can limit the absorption of other nutrients. The tannin content in commonly cultivated FB seeds ranges from 5 to 10 g/kg of dry matter, although there are also low-tannin (with tannin content below 1.0%) and tannin-free faba beans varieties [3, 4, 5]. As enzyme inhibitors, tannins can form a tannin-enzyme (protein) complex, which has been well investigated and documented in *in vitro* studies. Inhibition of digestive enzymes, i.e. trypsin, α-amylase, and lipase, has also been reported [4]. Thus, tannins in the diet trigger numerous nutritional problems. They reduce feed consumption, growth, egg weight, or nitrogen and protein digestibility in chickens [6]. By influencing the mineral availability and absorption (including Ca, P, Mg, Na, K, and Fe), tannins can alter the bone formation process [7]. In addition, other effects include hepatotoxicity, toxic nephrosis, and damage to intestinal mucosa [8]. Moreover, although the activity of lectins in FB seeds is low, the compounds bind to the receptors of epithelial cells of the intestinal mucosa, damage mucosal cells, and disturb digestive processes [9]. Lectins also change gut immune function, decrease the production of endocrine cells and gut hormones, and interfere with bacteria in the gut lumen [10]. On the other hand, health-enhancing properties of FB seeds such as antibacterial, antiparasitic, antioxidant, antigenotoxic, antidiarrheal, and anticarcinogenic activities have been reported [8]. Furthermore, FB seeds (irrespective of the tannin concentration) include approx. 7.3–9.9% (73–99 g/kg) of crude fiber, which contains 85% of non-starch polysaccharides [5]. Studies conducted in broilers have shown that low-fiber diets influencing the transit time increase the incidence of enteric disorders [11]. Furthermore, dietary fiber, also that from FB seeds, can increase loss of minerals, e.g. Zn, Ca, Mg, Fe, and Cu [12]. On the other hand, the total production of short chain fatty acids e.g. butyrate having important implications for metabolism as well as the structure and function of intestinal epithelial cells depends on the amount of dietary fiber in the diet. The content and type of dietary fiber are also responsible for changes in the secretion of gastric and pancreatic juices [12]. Moreover, lower serum concentrations of triglycerides, total cholesterol, and phospholipids in the high-density lipoprotein fraction are observed in chickens when a high level of fiber is provided in the diet. Lipids in low-density lipoproteins are not affected by the treatment [13].

Based on the growth rate and feed efficiency in FB fed birds, an inclusion up to the level of 200 g of FB seeds/kg of diet in broiler chickens has been recommended [3, 14, 15]. The discrepancy observed between studies may reflect differences in cultivars or feed formulation strategies, and especially failure to balance available energy and inappropriate content of digestible amino acids in the feed [3, 14]. However, the overall effects of dietary FB depend both on the species, genotype, and age of birds and on the FB cultivar. Considering the main negative effects of anti-nutritive factors on mineral utilization and function of the digestive tract, it is expected that diet introducing raw low-tannin FB seeds can negatively influence bone structure and gut development. Numerous studies have shown the feeding value of FB for poultry, but reports on the effects on the gut-bone axis are not commonly available. There are no experiments focused on the gut-bone axis in broiler chickens. Therefore, the growing interest in using FB seeds has inspired us to study the effects of the replacement of soybean meal with low-tannin FB seeds on the gut-bone axis. The aim of the study was to examine the influence of different concentrations of raw low-tannin FB seeds on the gut-bone axis in Ross 308 broilers. Serum lipidogram was the criterion in assessment of the FB effects on metabolism. A possible damaging effect of raw low-tannin FB seeds was monitored by determination of the activity of selected indicator enzymes (ALAT and AspAT). To our knowledge, there is no information on the influence of dietary manipulation on gut and bone development or the physiological and histological consequences in broiler chickens fed diets containing raw low-tannin FB seeds in different concentrations in the starter and grower diet, respectively. Thus, the present experiment was performed to evaluate these effects.

## Materials and methods

The experimental procedures and protocols used throughout this study were approved by the Local Ethics Committee for Animal Experimentation in Lublin (until February 1, 2016 it operated under the name of The Second Local Ethics Committee on Animal Experimentation of University of Life Sciences in Lublin), Lublin, Poland (resolution No. 72/2017).

### Experimental design, birds and diets

In total, 96 1-day-old Ross 308 broiler chickens obtained from a commercial hatchery with an initial body weight of approx. 45.2±0.28 g were used in the study. The birds were randomly selected to one of the 3 dietary treatments with 32 chickens in each divided into 8 pens with 4 birds per each pen. All birds were held in battery cages located in an environmentally controlled room under standard rearing conditions. The air temperature set at the optimal level depended on the age. The initial temperature (33°C) was reduced by 2°C weekly until the final value of 24°C. The birds had constant access to fresh water and were fed *ad libitum* in accordance with the stage of the production (Table 1): starter in the form of crumble (1–21 days) and grower in the loose form (22–35 days). The nutrient composition of the basal diet was analyzed using standard methods (Table 1) [16, 17, 18, 19].

**Table 1:** Feed ingredients and nutritive value of the diet.

Item	Starter			Grower		
	control	group I	group II	control	group I	group II
Ingredient (%)						
Maize	49.92	44.59	39.37	55.285	45.245	41.22
Faba bean	-	8.00	16.00	-	15.00	22.00
Soybean meal	41.50	38.00	34.50	36.00	29.50	26.00
Rapeseed oil	4.70	5.50	6.20	5.00	6.50	7.00
L-lysine	-	-	-	0.02	0.02	0.02
DL-methionine	0.22	0.24	0.26	0.195	0.225	0.24
Limestone	1.30	1.33	1.36	1.31	1.35	1.37
Monocalcium phosphate	1.50	1.48	1.45	1.32	1.285	1.27
Salt	0.36	0.36	0.36	0.37	0.375	0.38
Premix*	0.50	0.50	0.50	0.50	0.50	0.50
Total	100.00	100.00	100.00	100.00	100.00	100.00
The nutritional value of mixtures:						
ME, MJ	12.77	12.77	12.75	13.08	13.08	13.05
Crude protein, %	22.3	22.3	22.3	20.3	20.3	20.3
Crude fibre, %	2.601	2.940	3.281	2.532	3.168	3.464
Lysine, %	1.291	1.311	1.332	1.170	1.209	1.216
Methionine, %	0.578	0.582	0.587	0.528	0.529	0.529
Methionine + cysteine, %	0.973	0.967	0.961	0.895	0.877	0.866
Threonine, %	0.919	0.911	0.903	0.836	0.821	0.808
Tryptophan, %	0.292	0.285	0.278	0.262	0.249	0.241
Ca, %	0.979	0.982	0.982	0.926	0.925	0.924
P, %	0.699	0.704	0.707	0.642	0.652	0.656
P available, %	0.456	0.456	0.454	0.405	0.406	0.405
Na, %	0.167	0.165	0.164	0.170	0.169	0.169

*—1 kg of mixtures contained (starter/grower): vitamins A– 13500/10000 IU, D3 − 10000/3000 IU, E– 80/50 mg, K– 4/3 mg. B1 − 3/2 mg, B2 − 8.75/7 mg, B6 − 5/4 mg, B12 − 24/27.5 μg, PP– 70/70 mg, B5 − 25/14 mg, B9 − 2.00/1.50 mg, H– 0.20/0.15 mg, Bt− 500/500 mg; microelements: Fe– 80/80 mg, Mn– 100/100 mg, Zn– 80/60 mg, Cu– 9/8 mg, I– 1.25/1.00 mg, Se –0.275/0.25 mg, Co– 0.30/0.25 mg, Ca– 1.311/1.28 g, coccidiostatic, antioxidant.

All the birds were divided randomly into 3 groups depending on the amount of low-tannin FB seeds (*cv*. Alba, with white colour flowers and a seed cover of clear beige) introduced into the starter/grower mixtures in isonitrogenous and isoenergetic diet, according to the system: the control group was fed standard diet with soybean meal and without FB seeds, group I was fed 8%/15% of low-tannin FB seeds, and group II was fed 16%/22% of low-tannin FB seeds. Faba bean seeds introduced into the starter/grower mixtures replaced soybean meal protein in the amount of 10/20% or 20/30%, respectively. In the preliminary experiment it was found that the inclusion of FB seeds in that amount did not alter broiler chickens body weight (data not shown).

During the study, the chickens were weighed and feed consumption was monitored. These data were used to calculate the body weight gain, feed intake, and feed conversion ratio.

At the end of the experiment, 1 bird randomly selected from each pen (8 birds from each group) was weighed, stunned by mechanical methods and then decapitated. Ten hours before the slaughter, the birds were starved, and only access to water was unlimited. Blood samples were collected during the slaughter.

### Feed analysis

Dry matter, crude protein, and crude fiber in the diet were determined according to AOAC International [16] procedures No. 934.01, 942.05, and 978.10, respectively.

Total phosphorus was determined colorimetrically [16]. The Ca content in the feed samples was determined using the FAAS technique [16]. The composition of amino acids in the dietary mixture was determined by ion-exchange chromatography as described previously [17]. Cysteine and methionine were determined in a separate analysis. Assimilable lysine was determined based on the difference between total lysine and so-called residual lysine, which did not react with dinitrofluorobenzene [17].

The content of the most important anti-nutritional factors in FB seeds was analyzed: tannins with the colorimetric method [18] and trypsin inhibitors with the method described by Kakade et al. [19].

The FB seeds (low-tannin variety) contained 87% of dry matter, 25.41% of crude protein, 8.23% of crude fiber, 0.42% of tannins, and 0.15% of trypsin inhibitors. The quantity of tannins was in the range for the low-tannin variety specified in the literature (below 1%) [3, 4, 5]. This quantity was lower by approx. 60% than in the high-tannin FB cultivar (*cv*. Granite), in which tannins are present in the range of approx. 1.02% [20].

### Hematological and serum biochemical analyses

After clotting at room temperature, the blood samples were centrifuged and serum was frozen at -80°C for further analysis. Other blood samples were transferred to a glass tube containing EDTA and mixed with it. The assay of the blood samples was carried out in the laboratory soon after the blood was successfully collected. Manual counting of total red and white blood cells was carried out. The hemoglobin concentration (Hb) and packed cell volume (PCV) were determined on a hematological analyzer ABACUS Junior Vet (Diatron, Vienna, Austria).

Total protein, glucose, uric acid, creatinine, lipid profile (total cholesterol, HDL—high density lipoproteins, LDL—low density lipoproteins, TG—triglycerides), alanine aminotransferase (ALAT), aspartate aminotransferase (AspAT), lactate dehydrogenase (LDH), amylase (AMY), iron (Fe), calcium (Ca), and phosphorus (P) were determined with the colorimetric method using a Mindray BS-120 biochemical analyzer (Mindray Bio-Medical Electronics Co., Ltd, Shenzhen, China) and sets of ready-made biochemical reagent kits (Alpha Diagnostics, Warsaw, Poland) [17].

### Tissue collection and histomorphometrical analysis

#### Intestinal tract analysis

Two segments of the intestine (10 mm long each) from the duodenum (10 mm distal to the pylorus) and jejunum (50% of the total length) were taken from each bird, opened along the mesenteric border, and pinned flat without stretching on histological cassettes in physiological saline [21]. The liver samples were collected from the same lobe from each bird. The samples were fixed in 4% buffered formaldehyde (pH 7.0) for 24 h, dehydrated in graded ethanol solutions, and then fixed and embedded in paraffin in a STP 120 spin tissue processor (Waltham, MA, USA). Further, 4-μm thick tissue samples cut with a microtome (Microm HM 360, Microm, Walldorf, Germany) were subjected to histology as described previously [21, 22, 23]. Masson’s trichrome staining was used to differentiate the small intestine wall layers more efficiently [24].

A microscope (AXIOVERT 200M, Carl Zeiss, Jena, Germany) and a camera (AxioCam HRc; Carl Zeiss, Jena, Germany) were used to collect microscopic (two-dimensional) images. Objective magnification of 4x, 10x, 20x, and 40x was used to collect images of the different intestinal structures. The structure of the liver tissue and small intestine wall was examined by microscopic observation and using graphic analysis software (Olympus cellSens Version 1.5; Olympus, Tokyo, Japan) [25, 26].

The following morphometric parameters in the intestine were analyzed: the thickness of mucosa, submucosa, and myenteron (longitudinal and transversal lamina); villus epithelium thickness, crypt depth (defined as the depth of the invagination between adjacent villi from the bottom of the crypt to the base of villi); crypt width (measured in the middle of the crypt depth); the number of crypts (active: showing mitoses and Paneth cells, having an open internal space and access to the intestinal lumen; inactive: showing no mitoses and Paneth cells, having a closed internal space; total: inactive plus active crypts); villus length (from the tip of the villi to the villus-crypt junction); villus thickness (measured in the middle of villus height); the number of villi; and the small intestine absorptive surface [26]. Only vertically oriented villi and crypts were measured.

The microscopic observations also allowed identification and assessment of normal structures such as portal triads and terminal hepatic venules, necessary for the evaluation of the lobular architecture and small hepatocytes (a characteristic of regeneration). Moreover, the following parameters were analyzed: the total number of cells/mm^2^; total hepatocyte number/mm^2^; the number of non-hepatocyte cells/mm^2^; the number of hepatocyte nuclei/mm^2^; the number of mononuclear hepatocytes/mm^2^; and the number of multinucleated hepatocytes/mm^2^ [27, 28].

Immunohistochemical reaction with a mouse monoclonal antibody to the 200-kD neurofilament heavy subunit neuronal marker (Sigma-Aldrich, St. Louis, MO, USA, dilution 1:40) was performed to localize Meissner and Auerbach plexuses. After deparaffinizing and rehydration, antigen retrieval was achieved by boiling sections of the duodenum and jejunum in citrate buffer (pH 6.0) three times for 5 minutes in a microwave oven (700 W). A 3% solution of hydrogen peroxide in methanol (1:1) was used for 30 minutes to block endogenous peroxidase activity. After blocking in normal serum, the sections were incubated with the first antibody at 4°C overnight and then incubated (30 minutes) with the second antibody: biotinylated rabbit polyclonal to mouse immunoglobulin (Sigma-Aldrich, St. Louis, MO, USA, dilution 1:200) with streptavidin/HRP (DacoCytomation, Glostrup, Denmark, dilution 1:300) and developed in 3`3`-diaminobenzidine tetrahydrochloride (DAB; DacoCytomation, Glostrup, Denmark) for 15 minutes at room temperature. Negative control sections for immunohistochemical staining were prepared by omission of the primary antibody; next, all the samples were counterstained with Mayer’s hematoxylin.

Microscopic images of the immunohistochemistry reactions described above were subjected to further analysis and the variables analyzed for neurofilament detection included: the cross section area of the nerve ganglion; sphericity, perimeter, mean Feret diameter, and the minimal and mean diameter of the ganglion. The Feret diameter was defined as the distance between two parallel planes restricting the object perpendicular to that direction [29].

#### Bone analysis

Immediately after slaughter, tibiae from individual chickens were dissected, cleaned from the remnants, weighed, and wrapped in gauze soaked in isotonic saline and kept frozen at -25°C until further examination. The bone mechanical properties were determined after overnight thawing at room temperature using the three-point bending test of the bone mid-diaphysis on a Zwick Z010 universal testing machine (Zwick GmbH & Company KG, Ulm, Germany) [30]. The load was applied in the anterior-posterior plane of the bone with a displacement rate of 10 mm/min until fracture [31]. Moreover, the cortical cross section area, cortical index, vertical cortical index, second (cross-sectional) moment of inertia, and radius of gyration about the medial-lateral axis were calculated as previously [32, 33]. The following mechanical properties were determined: yield strength, ultimate strength, elastic stress, bending moment, elastic energy, work to fracture, Young modulus, elastic strain, ultimate strain, and toughness modulus [34, 35]. All mechanical properties were determined using Origin software (OriginLab, Northampton, MA, USA).

After evaluation of the geometric and mechanical properties, the bones were defatted and dried at 105°C to constant weight to determine the bone tissue density of the midshaft using a gas pycnometer (AccuPyc 1330, Micromeritics, Nocross, GA, USA) [36]. Finally, the bones were ashed in a muffle furnace at 500°C and the bone ash percentage was determined. The content of selected mineral components (Ca, P) in the bone was assessed by atomic absorption spectrometry using a Unicam 939/959 (Unicam, Kassel, Germany) apparatus [16]. The content of Ca, P in the bone was expressed as the content of these components in crude ash.

### Statistical analysis

Data were analyzed using Statistica 12 software (StatSoft, Inc., Tulsa, OK, USA). The distribution of variables was tested for normality using the Shapiro–Wilk test and equality of variance was tested with the Brown-Forsythe test. In the next step, the data were analyzed by One Way ANOVA followed by Tukey’s HSD post-hoc test. For all tests, P<0.05 was considered statistically significant. The data are presented as means with their standard deviations (SD).

## Results

### Rearing results of broiler chickens, liver weight

The final body weight, body weight gain, total feed intake, and total feed conversion ratio did not differ between all the groups (Table 2).

**Table 2:** Body weight and rearing results of broiler chickens.

Item	control	group I	group II
Final body weight (35^th^ day), g	1919±55^a^	1883±35^a^	1879±44^a^
Body weight gain (1–35 days), g	1852±30^a^	1833±34^a^	1829±44^a^
Total feed intake (1–35 days), g	3000±65^a^	2940±22^a^	2960±77^a^
Total feed conversion ratio (1–35 days), g	1590±42^a^	1604±31^a^	1620±85^a^

Data are mean ± SD.

^a^—mean values in rows with different letters differ significantly at P<0.05.

control—the control group fed standard diet without faba bean,

group I—the group fed 8%/15% (starter/grower) of low-tannin faba bean seeds,

group II—the group fed 16%/22% (starter/grower) of low-tannin faba bean seeds.

The birds fed the diet containing the higher amount of low-tannin FB seeds (group II) had heavier livers compared to the control group (Table 3).

**Table 3:** Effect of faba beans on the absolute and relative weight of liver from broiler chickens at the age of 35 days.

Item	control	group I	group II
Absolute weight, g	33.8±3.1^a^	35.5±4.1^a^^b^	39.2±4.7^b^
Relative weight, %	1.8±0.24^a^	1.9±0.14^a^^b^	2.1±0.3^b^

Data are mean ± SD,

^a, b^–mean values in rows with different letters differ significantly at P<0.05.

Description of the experimental groups as in Table 2

### Hematological parameters and blood serum biochemistry

The intake of low-tannin FB seeds, irrespective of their amount, did not influence the basal hematological parameters (Table 4).

**Table 4:** Effect of faba beans on basal hematological parameters in blood from broiler chickens at the age of 35 days.

Item	control	group I	group II
RBC, 10^12^/L	1.85±0.12^a^	1.87±0.13^a^	1.92±0.14^a^
WBC, 10^9^/L	20.20±2.1^a^	19.11±1.0^a^	20.40±1.6^a^
Hb, mmol/L	7.69±0.20^a^	7.60±0.36^a^	7.80±0.43^a^
PCV, L/L	0.25±0.02^a^	0.25±0.02^a^	0.24±0.03^a^

Data are mean ± SD; RBC—red blood cells; WBC—white blood cells; Hb—hemoglobin; PCV—packed cell volume.

^a^–mean values in rows with different letters differ significantly at P<0.05.

Description of the experimental groups as in Table 2.

The concentration of total cholesterol and calcium decreased in the groups fed low-tannin FB seeds, irrespective of their amount in the diet (Table 5). The concentration of TG decreased only in group I, compared to the control group. The lowest concentrations of uric acid and P were detected in group II; it differed significantly from the highest concentration noted in the control group (Table 5). The activity of AspAT increased in group II, compared to the other groups. No other alterations in serum parameters were observed.

**Table 5:** Effect of faba beans on blood serum parameters in broiler chickens at the age of 35 days.

Item	control	group I	group II
Total cholesterol, mmol/L	3.65±0.12^b^	2.90±0.41^a^	3.22±0.11^a^
LDL, mmol/L	0.80±0.22^a^	0.58±0.16^a^	0.66±0.11^a^
HDL, mmol/L	1.89±0.91^a^	2.20±0.49^a^	2.23±0.29^a^
TG, mmol/L	0.49±0.12^b^	0.32±0.06^a^	0.44±0.02^a^^b^
ALAT, U/L	11.12±3.53^a^	10.82±3.92^a^	13.44±4.21^a^
AspAT, U/L	381±33^a^	382±61^a^	622±146^b^
LDH, U/L	1776±715^a^	2203±1442^a^	2451±883^a^
AMY, U/L	649±62^a^	459±63^a^	544±38 ^a^
Creatinine, μmol/L	15.2±3.1^a^	12.4±1.4^a^	12.7±1.4^a^
Glucose, mmol/L	12.3±1.1^a^	13.2±2.9^a^	12.6±0.6^a^
Total protein, g/L	25.0±4.1^a^	25.0±3.2^a^	28.2±1.2^a^
Uric acid, mmol/L	0.31±0.08^b^	0.24±0.04^a^^b^	0.19±0.06^a^
Ca, mmol/L	2.26±0.08^b^	1.88±0.13^a^	1.94±0.12^a^
P, mmol/L	2.55±0.32^b^	2.15±0.17^a^^b^	2.03±0.28^a^
Fe, μmol/L	16.5±3.0^a^	15.0±3.0^a^	16.2±3.1^a^

Data are mean ± SD,

^a, b^–mean values in rows with different letters differ significantly at P<0.05;

AMY—amylase; ALAT—alanine aminotransferase; AspAT—aspartate aminotransferase; LDH—lactate dehydrogenase; TG—triglycerides; HDL—high density lipoproteins, LDL—low density lipoproteins; Ca—calcium; P—phosphorus; Fe—iron.

Description of the experimental groups as in Table 2.

### Gastro-intestinal tract morphology

The intake of low-tannin FB seeds resulted in a narrower longitudinal myenteron in the duodenum in group I, compared to the other birds (Table 6). The transversal lamina thickness was reduced in both experimental groups, compared to the controls. The intake of low-tannin FB seeds led to reduction of the submucosa in group I and widening was observed in group II, compared to the controls. The mucosa thickness and villus length decreased depending on the amount of low-tannin FB seeds, the villus thickness increased depending on the amount of low-tannin FB seeds, and the villar epithelium thickness increased, irrespective of the low-tannin FB seeds amount, compared to the control group. The total number of villi decreased in group II, compared to the control group, but the crypt depth increased in both groups fed low-tannin FB seeds. However, the intestine absorptive surface in the duodenum decreased in both groups depending on the amount of FB seeds (Table 6).

**Table 6:** Effect of faba beans on histomorphometric parameters of the duodenum in broiler chickens at the age of 35 days.

Item	control	group I	group II
Myenteron thickness, μm			
Longitudinal lamina	65.8±12.1^b^	37.5±15.1^a^	62.1±17.6^b^
Transversal lamina	133±34^b^	112±9^a^	105±29^a^
Submucosa thickness, μm	36.9±6.9^b^	32.0±4.5^a^	51.2±11.1^c^
Mucosa thickness, μm	1489±175^c^	1351±259^b^	1183±117^a^
Villar epithelium thickness, μm	13.6±4.2^a^	23.6±3.9^b^	24.1±7.4^b^
Villus length, μm	1065±136^c^	802±109^a^	914±78^b^
Villus thickness, μm	65.4±9.6^a^	89.9±9.7^c^	74.3±9.2^b^
Total number of villi/mm	11.0±2.6^b^	9.3±1.6^a^^b^	7.2±1.1^a^
Crypt depth, μm	88±22^a^	134±42^b^	154±39^c^
Crypt width, μm	38.2±6.5	40.9±8.1	40.4±8.6
Intestine absorptive surface, μm^2^	26.6±4.3^c^	17.6±3.1^a^	21.4±3.4^b^
Number of crypts/mm			
Active crypts	2.5±1.1^a^	4.0±1.4^a^	3.8±1.1^a^
Inactive crypts	15.2±3.3^a^	17.7±4.3^a^	13.2±2.7^a^
Total	17.3±3.4^a^	21.7±4.8^a^	16.9±3.0^a^

Data are mean ± SD,

^a, b, c^–mean values in rows with different letters differ significantly at P<0.05.

Description of the experimental groups as in Table 2.

The intake of low-tannin FB seeds led to widening of the longitudinal myenteron and submucosa in the jejunum in the birds from group II, compared to the other groups (Table 7), while the reduction of submucosa and villar epithelium thickness was observed in group I. The intake of low-tannin FB seeds resulted in shorter villi and reduction of villus thickness and crypt depth and width in group I, compared to the other birds (Table 7). On the other hand, the villus thickness and total number of villi were reduced in group II. Furthermore, the intake of low-tannin FB seeds resulted in an increase in the crypt depth in group II, compared to the other groups. Although of the number of active crypts did not change, the number of total and inactive crypts decreased in group II, compared to the other groups (Table 7). Furthermore, the intake of low-tannin FB seeds influenced Auerbach plexuses in both parts of the intestine in group II, while all the parameters of Meissner plexuses increased only in the jejunum in both groups of birds fed low-tannin FB seeds (Tables 8 and 9).

**Table 7:** Effect of faba beans on histomorphometric parameters of the jejunum in broiler chickens at the age of 35 days.

Item	control	group I	group II
Myenteron thickness, μm:			
Longitudinal lamina	31.4±6.8^a^	30.8±6.5^a^	60.4±10.5^b^
Transversal lamina	106±13^a^	111±9^a^	106±21^a^
Submucosa thickness, μm	32.9±7.8^b^	20.6±4.1^a^	49.1±10.0^c^
Mucosa thickness, μm	1412±182^b^	1450±329^b^	1254±92^a^
Villar epithelium thickness, μm	31.5±7.6^b^	24.5±5.5^a^	40.5±7.7^c^
Villus length, μm	1078±203^b^	893±147^a^	1085±92^b^
Villus thickness, μm	109±17^c^	67.4±6.2^a^	76.9±16.8^b^
Total number of villi/mm	11.6±1.0^b^	13.0±3.1^b^	8.2±0.8^a^
Crypt depth, μm	99±33^b^	76±26^a^	177±32^c^
Crypt width, μm	42.8±10.2^b^	33.4±7.4^a^	41.5±8.4^b^
Intestine absorptive surface, μm^2^	21.9±5.3^a^	23.8±8.5^a^	22.6±4.3^a^
Number of crypts/mm			
Active crypt	4.9±1.5^a^	4.9±1.3^a^	5.9±1.0^a^
Inactive crypt	11.9±2.6^b^	11.4±3.5^b^	6.6±1.7^a^
Total	16.8±2.8^b^	16.3±2.9^b^	12.5±1.6^a^

Data are mean ± SD;

^a, b, c^–mean values in rows with different letters differ significantly at P<0.05.

Description of the experimental groups as in Table 2.

**Table 8:** Effect of faba beans on histomorphometric parameters of nerve plexuses in the duodenum in broiler chickens at the age of 35 days.

Item	control	group I	group II
*Auerbach plexus*			
Area, μm^2^	2856±1844^a^	2874±1335^a^	4780±2824^b^
Perimeter, μm	224±99^a^	241±85^a^^b^	315±140^b^
Mean Feret diameter, μm	67.5±29.2^a^	72.4±25.0^a^^b^	94.3±42.0^b^
Min. diameter, μm	41.4±12.3^a^	38.5±10.9^a^	44.6±9.5^a^
Mean diameter, μm	55.5±15.2^a^	55.7±11.7^a^	69.2±17.6^b^
Sphericity	0.342±0.209^a^	0.278±0.202^a^	0.222±0.135^a^
*Meissner plexus*			
Area, μm^2^	1295±711^a^	1175±821^a^	1108±610^a^
Perimeter, μm	174±66^a^	173±68^a^	164±65^a^
Mean Feret diameter, μm	54.5±19.3^a^	52.1±20.3^a^	49.3±19.5^a^
Min. diameter, μm	20.2±4.7^a^	17.7±6.2^a^	18.2±4.6^a^
Mean diameter, μm	35.4±8.5^a^	32.4±9.9^a^	32.4±8.0^a^
Sphericity	0.125±0.090^a^	0.106±0.103^a^	0.118±0.088^a^

Data are mean ± SD;

^a, b^–mean values in rows with different letters differ significantly at P<0.05.

Description of the experimental groups as in Table 2.

**Table 9:** Effect of faba beans on histomorphometric parameters of nerve plexuses in the jejunum in broiler chickens at the age of 35 days.

Item	control	group I	group II
*Auerbach plexus*			
Area, μm^2^	1594±1140 ^a^	2424±2176 ^a^	2705±2376 ^a^
Perimeter, μm	193±115^a^	237±140^a^	222±117^a^
Mean Feret diameter, μm	56.9±33.1^a^	70.9±41.3^a^	66.2±34.4^a^
Min. diameter, μm	25.1±7.0^a^	28.5±8.6^a^^b^	35.3±14.1^b^
Mean diameter, μm	39.0±8.1^a^	46.3±15.1^a^^b^	50.8±21.6^b^
Sphericity	0.232±0.168^a^	0.213±0.189 ^a^	0.287±0.218^a^
*Meissner plexus*			
Area, μm^2^	275±235^a^	1242±690^b^	1207±571^b^
Perimeter, μm	87.5±57.7^a^	198.7±79.5^b^	159.8±40.8^b^
Mean Feret diameter, μm	25.8±16.7^a^	59.7±23.3^c^	47.7±12.4^b^
Min. diameter, μm	7.3±2.3^a^	16.8±3.6^b^	21.9±6.4^c^
Mean diameter, μm	14.9±4.7^a^	32.4±7.2^b^	35.4±7.8^b^
Sphericity	0.145±0.139^b^	0.063±0.041^a^	0.162±0.099^b^

Data are mean ± SD;

^a, b, c^–mean values in rows with different letters differ significantly at P<0.05.

Description of the experimental groups as in Table 2.

The intake of low-tannin FB seeds did not change the number of total and mononuclear hepatocytes or the number of total hepatocyte nuclei, irrespective of the amount of the seeds, while the number of total cells increased only in group I. The number of non-hepatocyte cells increased in both groups fed low-tannin FB seeds. On the other hand, multinuclear hepatocytes increased in group I, compared to group II (Table 10).

**Table 10:** Effect of low-tannin faba beans on histomorphometric parameters of liver in broiler chickens at the age of 35 days.

Item	control	I group	II group
Total cells	11095±1148^a^	13619±1118^b^	12340±948^a^^b^
Total hepatocytes	9340±701^a^	9346±1133^a^	8759±1068^a^
Total hepatocyte nuclei	9553±856^a^	9670±720^a^	8905±842^a^
Mononuclear hepatocytes	9127±693^a^	9020±650^a^	8580±735^a^
Multinuclear hepatocytes	213±35^a^^b^	320±188^a^	163±61^b^
Non-hepatocyte cells	1665±278^a^	4273±681^b^	3581±1138^b^

Data are mean ± SD;

^a, b^–mean values in rows with different letters differ significantly at P<0.05.

Description of the experimental groups as in Table 2.

### Histopathological analysis of the liver tissue

**Fig 1:**
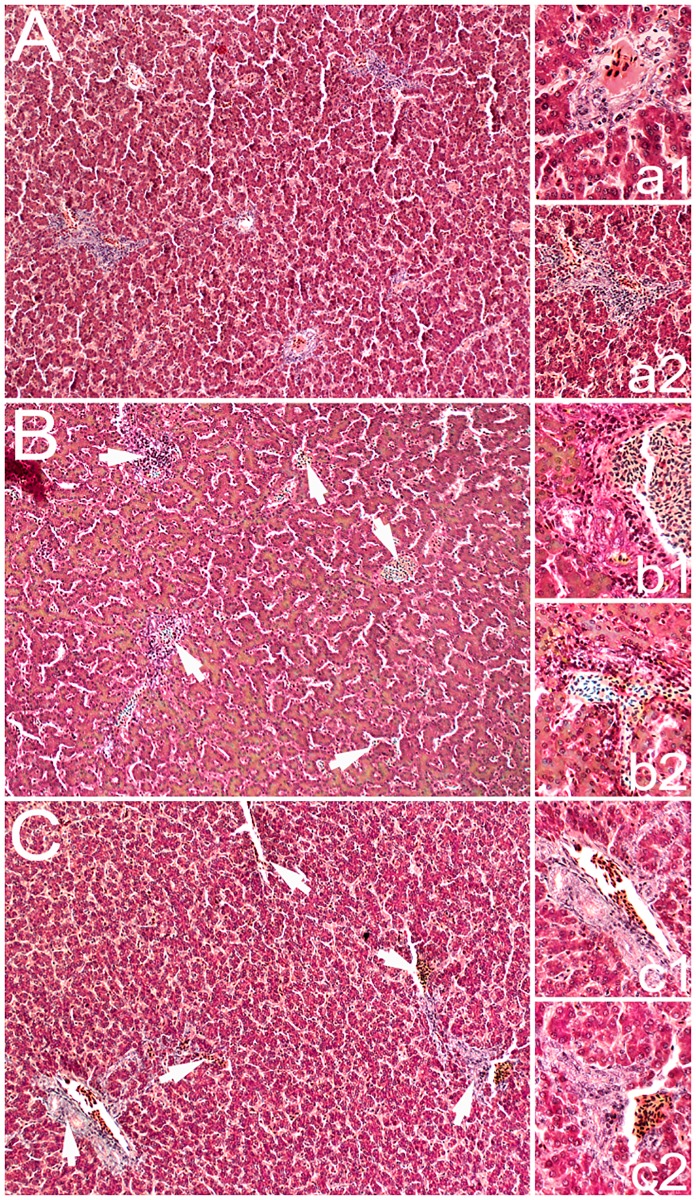
Representative images of MT staining carried out on formaldehyde-fixed sections from liver tissue of chickens fed different amounts of faba beans seeds introduced into the starter/grower mixtures. A—control group; B—group I fed 8%/15% (starter/grower) of low-tannin FB seeds; C—group II fed 16%/22% (starter/grower) of low-tannin FB seeds. No local lymphocytic infiltrates in portal tracts and sinusoids are present in the control group (a1, a2). Leukemic lymphocytic infiltrates (indicated by white arrows) are present in the portal tracts and sinusoids of livers sampled from the birds in group I (b1, b2) and group II (c1, c2). Magnification x200.

The histological examination revealed no marked changes or degenerative changes in the control group. Normal structure of liver parenchyma with preserved portal and triad architecture was observed. On the other hand, the intake of low-tannin FB seeds resulted in focal and intense inflammatory changes with dose-dependent severity (Fig 1). Animals fed the lower doses of low-tannin FB seeds had greater and more numerous inflammation foci, where periportal inflammatory cell infiltrate was observed. However, there were no marked signs of edema or hyperemia in any group.

### Bone morphology, mechanical and geometric parameters

The intake of low-tannin FB seeds resulted in reduction of the bone weight in group II, compared to the control, simultaneously not influencing the length (Table 11). The consumption of low-tannin FB seeds reduced bone geometric properties depending on their amount in the diet. On the other hand, an increase in bone mechanical properties was observed in group I, compared to the controls except the ultimate strain, which increased in group II, compared to group I (Table 11). However, the value of yield and ultimate strengths decreased in group II, compared to the control group.

**Table 11:** Effect of faba beans on basal morphology, mechanical and geometric parameters of the tibia in broiler chickens at the age of 35 days.

Item	control	group I	group II
Bone general properties			
Bone weight, g	17.5±1.2^b^	16.0±1.8^a^^b^	15.2±1.9^a^
Bone length, mm	107±3^a^	103±2^a^	102±2^a^
Bone weight/bone length, g/mm	0.171±0.017^a^	0.155±0.016^a^	0.153±0.019^a^
Bone geometric properties			
Cross section area, mm^2^	42.6±4.7^b^	34.3±5.5^a^	36.5±4.3^a^
Mean relative wall thickness	1.05±0.19^a^	0.95±0.35^a^	0.96±0.45^a^
Cortical index, %	50.0±4.1^b^	44.3±7.5^a^^b^	41.7±5.1^a^
Moment of inertia, mm^4^	200±47^b^	149±50^a^	173±38^a^^b^
Index of gyration, mm	2.17±0.13^a^	2.02±0.21^a^	2.22±0.24^a^
Bone mechanical properties			
Yield strength, N	184±18^b^	166±16^a^^b^	159±12^a^
Ultimate strength, N	332±19^b^	330±18^a^^b^	309±14^a^
Elastic stress, MPa	33.6±4.4^a^	48.5±14.2^b^	34.2±8.8^a^
Ultimate stress, MPa	72.0±116.5^a^	108.7±13.1^b^	73.6±13.6^a^
Bending moment, N·m	1.75±0.24^a^	1.70±0.15^a^	1.64±0.12^a^
Elastic energy, mJ	41.3±8.6^a^	43.8±11.0^a^	35.6±3.7^a^
Work to fracture, mJ	482±141^a^	471±131^a^	456±94^a^
Young Modulus, GPa	2.96±0.66^a^	4.14±1.43^b^	2.95±0.82^a^
Elastic strain, %	1.20±0.16^a^	1.12±0.19^a^	1.15±0.15^a^
Ultimate strain, %	5.55±0.76^a^^b^	5.03±0.76^a^	6.22±1.18^b^
Toughness modulus, mJ/mm^3^	2.72±0.75^a^	3.35±0.94^a^	3.09±0.42^a^

Data are mean ± SD;

^a, b^–mean values in rows with different letters differ significantly at P<0.05.

Description of the experimental groups as in Table 2.

### Bone ash, Ca and P content, and densitometric analysis

The intake of the higher amount of low-tannin FB seeds in the diet resulted in a higher P concentration and content of ash in the bone, an elevated ash weight-to-volume ratio, and increased bone tissue density in group II, compared to the control group (Table 12). Subsequently, the Ca/P ratio decreased in group II, compared to the controls. Moreover, the P concentration increased and the Ca/P ratio decreased in group I, compared to the control group (Table 12).

**Table 12:** Effect of faba beans on ash, Ca and P content, and densitometric parameters of the tibia in broiler chickens at the age of 35 days.

Item	control	group I	group II
Ash, %	34.8±4.0^a^	35.9±3.3^a^	40.3±2.6^b^
Ash weight/volume, g/cm^3^	0.87±0.09^a^	0.91±0.11^a^^b^	0.98±0.06^b^
Ca, g/kg	442±11^a^	447±12^a^	444±16^a^
P, g/kg	178±2^a^	206±4^b^	207±3^b^
Ca/P ratio	2.49±0.08^b^	2.17±0.04^a^	2.15±0.07^a^
Bone tissue density, g/cm^3^	1.74±0.07^a^	1.77±0.08^a^^b^	1.85±0.07^b^
Bone mineral density, g/cm^2^			
Whole bone	0.175±0.010^b^	0.171±0.012^b^	0.155±0.011^a^
Distal part	0.206±0.020^b^	0.200±0.029^a^^b^	0.175±0.019^a^
Midshaft	0.169±0.018^b^	0.165±0.015^b^	0.144±0.016^a^
Proximal part	0.195±0.025^a^	0.177±0.082^a^	0.216±0.035 ^a^
Bone mineral content, g			
Whole bone	1.349±0.279^b^	1.214±0.276^a^^b^	1.047±0.234^a^
Distal part	0.366±0.087^b^	0.261±0.051^a^	0.240±0.052^a^
Midshaft	0.554±0.100^b^	0.493±0.122^b^	0.323±0.119^a^
Proximal part	0.196±0.073^a^	0.250±0.111^a^	0.253±0.086 ^a^

Data are mean ± SD;

^a, b^–mean values in rows with different letters differ significantly at P<0.05.

Description of the experimental groups as in Table 2.

The bone mineral density of the whole bone, distal part, and midshaft decreased in group II, compared to the other groups, except the bone mineral density in the distal part, which differed only from the control group. The bone mineral content of the whole bone decreased in group II, compared to the control group, but was significantly lower in the midshaft, compared to the controls and group I (Table 12). Moreover, the bone mineral content in the distal part of the tibia decreased in both groups fed low-tannin FB seeds, compared to the control group.

## Discussion

The limitation of the use of FB in poultry diet is mainly related to the presence of anti-nutritional factors that can depress poultry performance. The major factors are lectins, phytate, non-starch polysaccharides, amylase inhibitors, protease inhibitors (trypsin or chymotrypsin), and tannins. Moreover, the result of dietary replacement of soybean meal with low-tannin FB seeds depends not only on anti-nutritional factors but also on the species and age of birds and even on the genotypes of low-tannin FB [37].

The introduction of raw low-tannin FB seeds to the diet for broiler chickens, irrespective of their content (8/15% or 16/22% in the starter/grower mixture, respectively), yielded similar performance results in the present study (Table 2). Our findings are in agreement with the results of a study performed on male broiler chickens fed diet containing 0, 6, 12, and 18% of heated or raw FB for 42 days. There were no differences in the growth rate, fed intake, and feed conversion rate, irrespective of form FB used [3]. Another study has also shown no effect of dietary inclusion of FB in broiler chickens on the parameters of growth performance, feed intake, and mortality although, in contrast to our experiment, this study involved birds from 14 to 49 day of age [38]. Métayer et al. [39] have shown similar performance parameters in broiler chickens fed low-tannin FB seeds at 250 g/kg to those obtained in the control diet. Brévault et al. [40] have noted comparable effects of control diet in broiler chickens fed 200 g/kg of low-tannin FB. However, Perez-Maldonado et al. [41] have found that the inclusion of FB in poultry diets (310 g/kg) for 40 weeks did not affect body weight and mortality, but resulted in reduced hen-day egg production and egg weight. This is consistent with results obtained by Olaboro et al. [42], who have shown depressing effects of FB on the egg size. On the other hand, although no difference in daily feed intake and body weight gain was noted, the feed conversion ratio was higher in turkeys fed FB seeds for 56 days [43]. In contrast, Diaz et al. [44] have observed a reduction in body weight and a decrease in feed conversion efficiency in broilers fed extruded FB at 48% in the diet. A significant reduction in feed intake was observed in broilers fed 500 g/kg of high-tannin FB [45]. Another study has also documented a negative correlation with tannins when male broiler chickens were fed diet containing 500 g of raw FB with 9.9 g/kg of tannins [4]. No effect on the metabolism in chickens fed 300 g/kg of FB in the diet was reported by Jansman et al. [46].

It seems that the difference in this observation may have been be caused by the amount of the FB tested, which are bitter and thereby limit the feed intake. Thus, the different results related to the neutral, negative, or positive effects of FB depend mainly on tannins. Tannins from FB reduce not only feed intake in birds, but also protein digestibility by the formation of a protein-tannin complex [47]. However, there is a study showing that the digestibility of protein and amino acids increased in birds at the age of 30–50 days, irrespective of the presence of enzyme inhibitors [48].

In the current study, the tannin content in FB seeds was 0.42% of dry matter and, as mentioned above, there was no reduction of the fed intake and performance parameters. However, the parameters of poultry performance depend not only on the tannin concentration, but also on digestion of non-starch polysaccharides, changes in the viscosity of digesta in intestines, availability of digestive enzymes, and the amount of absorbed nutrients, which influence the growth, development, vital organ structure, and intestine function [48]. Thus, impaired body weight, fed intake, and feed conversion rate are results of changes in the intestine structure and function. To our knowledge, most of the reports on raw high-tannin FB seeds in poultry are focused on their influence on performance indices, and there are no studies on the effect of FB seeds, including low-tannin varieties, on intestine mucosa or liver tissue. Ortiz et al. [49] have shown a relationship between decreased body weight gain, fed intake, mortality, and histological changes in the intestines and liver in chickens caused by the addition of tannin-containing extracts of FB. Histological analysis has shown mainly structural alterations in the ileum and liver. Atrophy and shortening of villi as well as hyperplasia and hypertrophy of goblet cells have been observed as well. Histopathological alteration in the liver included extensive vacuolization of hepatocytes but without fat degeneration [49]. Vohra et al. [50] have reported thickening of mucosa in the esophagus and the crop in chickens fed diet containing 50 g of vegetable tannic acid/kg. Similar morphological alteration, mainly in the jejunum, has been observed by Rubio et al [51]. Besides shortening of villi and increased proliferation of enterocytes, an increase in intestinal transit has been noted in growing chickens fed raw FB at the dose of 500 g/kg. Moreover, the relative weight of the pancreas has been found to increase in birds fed diets containing 250, 350, or 500 g of raw FB seeds/kg; however, the intestinal tract of these chickens fed the lowest amount of FB seeds did not differ histologically from the control birds. Moreover, no alteration in the length of villi has been noted [51]. Another study has also demonstrated an increase in pancreas weight without changes in liver tissue in male broiler chickens fed diets containing vegetable tannins at the levels of 25 and 50 g/kg [52]. Sell et al. [53] have reported no histopathological alteration in the intestinal tract of chickens and laying hens, but the birds were fed high-tannin sorghum grains. However, the tannin concentration in high-tannin sorghum grains is similar to that present in FB seeds. In general, intestinal sections were found to be morphologically normal and only a slight reduction in the depth of the crypt and intestinal wall was noted.

Our study showed that the effect of inclusion of raw low-tannin FB seeds to poultry diet depended on their amount. The basal intestinal structures were significantly reduced in the birds fed the lower concentration of FB (8%/15% in the starter/grower, respectively), while an opposite effect was noted in the group fed low-tannin FB at 16% and 22% in the starter and grower, respectively. Similarly, the influence of low-tannin FB on the nerve plexuses in the intestine was dependent on the concentration used in the diet and additionally on the type of plexus and location in the intestinal tract. In general, enlargement of plexuses was observed it was approx. two-fold in Auerbach plexuses in the duodenum of birds fed the higher concentration of FB, and the Meissner plexus in the jejunum increased four times in both groups fed FB. Moreover, an increase in the number of non-hepatocyte cells in the livers of these chickens was observed (Fig 1). Further, the birds fed the lower doses of low-tannin FB seeds had more numerous and marked periportal inflammatory cell infiltrates (Fig 1Bb1 and 1Bb2).

Some studies prove that methionine limits the negative effect of tannins in the diet [54]. Sulphur-containing amino acids (methionine and cysteine) are present in a very low amount in FB seeds, but the seeds are rich in lysine [9]. For this reason, dietary mixtures should be isonitrogenous and isoenergetic. The diet of our chickens was also prepared in this way. However, the results of our study are in contrast with these findings, because it seems that the inclusion of low-tannin FB seeds in the diet of our broiler chickens, irrespective of their amount, resulted in a significant negative effect. Moreover, the histological observation carried out in our study revealed significant reduction of the absorptive surface of the duodenum. This was probably a result of the presence of anti-nutritional factors in FB, which can lead to decreased digestion. The greatest reduction of the absorptive surface of the duodenum was noted in the group fed the lower amount of low-tannin FB. It seems that the lower amount of FB caused more negative effects that the higher dose used in group II. It should be pointed out that the content of raw FB seeds in the diet of our chickens represented values permitted in poultry nutrition [16]. The alteration of the intestine wall depending on the amount of FB in the diet may have led to improper motor activity of the intestine. Additionally, the introduction of low-tannin FB to the diet may have influenced the intestinal flora and the host immune system due to the changes in the activity and number of crypts, where Paneth cells secrete antimicrobial peptides.

Our data have shown for the first time that low-tannin FB introduced into the diet at the concentration of 8%/15% or 16%/22% in the starter/grower mixture, respectively, can influence digestion, passage or absorption, and finally, general metabolism. There is very little information on the effect of low-tannin FB seeds on the liver metabolism and serum biochemical composition. Although the intake of low-tannin FB did not result in fibrosis of the liver, the increase in the number of non-hepatocyte cells in both groups might indicate that phagocytes (Kupffer cells) accumulated due to the inflammation caused by the anti-nutritional factors. The increase of multinuclear hepatocytes in the group fed the lower amount of FB might suggest enhanced metabolism in the liver. However, the birds fed the higher amount of low-tannin FB seeds had enlarged livers. This effect was observed previously, but the increase in the liver size in the chickens was also related to the intake of non-starch polysaccharides present in FB, leading to more enhanced activity of the liver, which produced more bile [55].

Our results have also shown that there was no influence of low-tannin FB on most of the analyzed serum parameters in the 35-day-old broiler chickens, except the changes in the concentration of total cholesterol and triglycerides, which decreased probably due to the higher amount of fiber in the experimental diets compared to the diet based on soybean meal only. Furthermore, the inclusion of low-tannin FB seeds in the diet of our chickens did not influence the activities of AspAT (except the group fed the higher amount of FB seeds), ALAT, and LDH. To our knowledge, the effect of the inclusion of low-tannin FB in the broiler diet on basal hematology or biochemical parameters has not been described; therefore, it is difficult to discuss our results.

However, contrary to mammals, the AspAT activity in birds is not specific for the liver, although it usually indicates liver or muscle damage. The diagnostic value of ALAT in poultry is limited [56]. Sometimes, birds with liver damage have ALAT in a normal range. A decrease in LDH can be related to liver disease or the breakdown of red blood cells [57]. Moreover, available data have indicated that the diet containing green beans reduced the blood cholesterol and triglyceride concentration in 35-day-old broiler chickens due to a high level of fiber [58]. The same effect of fiber on serum cholesterol has been reported by Burr et al. [59]. Other authors have also proved a cholesterol-lowering effect of different types of beans, which is attributed to an increase in rapid excretion of intestinal cholesterol and bile acids. An effect of green beans on ALAT and AspAT has not been reported [60, 61].

Nevertheless, both biochemical and hematological parameters are significant indicators of health status in all animals, including avian, and are an obligatory tool in the diagnosis and treatment of many diseases. Emiola et al. [62] have demonstrated that feeding mucuna bean (20%) reduces basal hematological and biochemical parameters in broiler chickens. The reduction of RBC, Hb, or PCV is a result of dysfunction of blood hematopoiesis, and the drop in WBC leads to dysfunction of defense capabilities of the organism. A decrease in serum total protein in birds is linked with inhibition of the utilization of protein in the intestinal tract. Additionally, hepatic enzymes increase due to elevated amino acid catabolism and liver damage [62]. Abbel-Monein [58] has reported that broiler chickens fed diet introducing 8%, 16%, and 24% of green beans for 35 days of their life do not exhibit changes in blood parameters or in blood glucose, total protein, and creatinine, which are in a normal range.

The inclusion of low-tannin FB seeds in the diet of our chickens did not influence the serum concentration of creatinine, but decreased uric acid. The serum creatinine concentration depends on the age, muscle mass, and kidney excretory capacity [63]. Serum urea acid increases as dietary nitrogen increases and can be used to assess amino acid utilization. Serum uric acid in birds is also influenced by the age, sex, reproductive state, and nutritional status [64, 65]. Tannins from FB can form a protein-tannin complex that reduces protein digestibility. Besides, depressed growth, death, hemolytic anemia, interference with mineral availability can be found in chickens fed plant diet containing anti-nutritional factors [66]. Tannins present in FB bind several macro elements e.g. calcium, phosphorus, or iron. Although, the serum iron concentration in our broiler chickens was unchanged and did not influence the red blood cell system, the serum concentration of calcium and phosphorus was decreased. However, the decreased serum calcium concentration was not linked with the bone calcium content. Similarly, the reduction of the serum phosphorus concentration did not result in a decrease in the bone phosphorus content.

As reported in many studies, anti-nutritive factors from plants can reduce absorption of calcium or phosphorus, thus influencing bone development [67]. Abbel-Monein [58] has reported that broiler chickens fed diet supplemented with green beans at a concentration of 8%, 16%, and 24% develop leg problems. Elkin et al. [68] have described leg abnormalities in broiler chickens fed high-tannin sorghum grains, caused by a change in the organic matrix, mainly in collagen synthesis [69]. Similar negative effects in broiler chickens at the age of 21 and 42 days of life fed high-tannin oak acorn (15%) were presented by Houshmand et al. [7]. Chickens’ tibiae had lower ash content and decreased mechanical strength, but without changes in their length or weight. In contrast, Moschini et al. [70] have described quite an opposite effect in chickens fed diet containing 25% or 50% of raw FB, as the birds exhibited no leg problems. Further, another study has shown that bone ash and bone mineralization in poultry are not influenced by tannic acid or tannins [54].

Our broiler chickens fed raw low-tannin FB seeds had no visible leg lesions or problems with locomotor function, but their tibiae were lighter, especially in the birds fed the higher amount of FB. The geometric analysis revealed reduction of the cross section area and wall thickness, indicating a decline of the bone midshaft, which influenced the densitometric parameters and the results of the mechanical tests.

All the results described above are consistent with the hypothesis of the gut-bone axis [32, 71, 72, 73], whose existence has been proved in several other experiments performed on both mammals and avian species [74, 75, 76, 77, 78]. Basal mineral elements and nutritional ingredients are absorbed in the digestive system. A correct structure and function of the intestine are needed for proper bone development ensuring adequate growth and locomotor activity. The gastrointestinal tract can act as a significant system influencing bone development and plays a crucial role in the process of skeleton mineralization.

It can be suggested that the inclusion of raw low-tannin FB seeds into the diet exerts an influence on the gastrointestinal tract mainly by decreasing the duodenal absorptive surface, probably in response to the anti-nutritive factors present in FB seeds. As a result, the proper bone metabolism could not be maintained. Therefore, although FB seeds can be a cheap source of protein, introduction thereof into poultry diet does not ensure comparable bone development as in the case of diet based on soybean meal only.

## Conclusion

The dietary inclusion of raw low-tannin FB seeds (8%/15% or 16%/22% in the starter/grower mixture, respectively) had no negative effects on broiler growth, but significantly disturbed the metabolism, intestine structure, and tibia characteristics. Thus, all these negative effects necessitate additional examinations before inclusion of FB seeds in poultry diet. The amount of low-tannin FB seeds that could be introduced into the diet of broiler chickens without disturbance in both the body weight gain and several gut-bone axis parameters is still under investigation.
